# Outcomes of Rectal Prolapse Using the Altemeier Procedure

**DOI:** 10.5812/ircmj.3666

**Published:** 2013-07-05

**Authors:** Seyed Mohsen Towliat, Shaban Mehrvarz, Hassan Ali Mohebbi, Ali Sate Bigdeli

**Affiliations:** 1 Research Center for Gastroenterology and Liver Disease, Baqiyatallah University of Medical Sciences, Tehran, IR Iran; 2 Trauma Research Center, Baqiyatallah University of Medical Sciences, Tehran, IR Iran; 3 Department of surgery Baqiyatallah University of Medical Sciences, Baqiyatallah University of Medical Sciences, Tehran, IR Iran

**Keywords:** Humeral Fractures, Proximal Humerus Fracture, Clavicle Fracture, Floating Arm

*Dear Editor,*

Rectal prolapse is a condition wherein the entire rectal wall protrudes through the anus; it can cause various functional difficulties that significantly compromise the patients’ quality of life. Progressive loss of function of the underlying pelvic structures (muscles and ligaments) results in fecal, urinary or concomitant urinary and fecal incontinence, pelvic pain, defecation disorders and rectal bleeding. Full thickness rectal prolapse does not respond to medical treatments and the definitive treatment for this condition is surgery. More than 100 surgical methods have been described for surgical treatment of this condition. However, a consensus has not been reached in selecting a preferred method of surgery. Therefore, method of choice is selected based on the surgeon’s experience and clinical findings (1-3). The surgeries are divided into abdominal or perineal. Perineal proctosigmoidectomy obviates the need for abdominal surgery. It, thus, is a preferred treatment procedure for high risk patients. However, the recurrence rate in this method is higher (4, 5). Since the abdomen is not opened and pelvic dissection isn’t performed, pelvic nerves aren’t manipulated and they remain intact. Consequently there is no risk of damage to these nerves or development of impotency (3). Abdominal surgery has the lowest recurrence rate but is associated with higher rate of complications, morbidity and mortality when compared to perineal surgery. In this retrospective study, we assessed Altemeier procedure for rectal prolapse evaluating the outcome and complications in patients who underwent this procedure at our hospital during 2001-2007. In preoperative evaluations, to rule out the presence of any associated lesions, rectal examination was performed.

In this method, patients are placed in prone position. Under general anesthesia an incision is made 1-2 cm above the pectinate line resecting mucosa and sub mucosa. The vessels are ligated. Muscular layers are orderly cut and at the end, the remaining segment of colon is sutured to the top of the pectinate line using absorbable material (polygalactin or vicryl sutures). If the patient has fecal incontinence along with rectal prolapse, posterior levatoroplasty (using plication technique) is also done during Altemeier procedure. Since the abdomen is not opened and pelvic dissection is not performed, pelvic nerves aren’t manipulated and they remain intact. Consequently there is no risk of damage to these nerves or development of impotency (3). The mean duration of follow up was 12 months. Fecal incontinence, constipation, diarrhea, assisting stool evacuation by hand is known to be the late complications that occur after a year following operation. Data regarding sex, age, signs, symptoms and complications were analyzed by SPSS software Ver.15 using t test and Chi Square test. In this study, 26 patients who underwent Altemeier procedure were evaluated. There were 8 females (30.8%) and 18 males (69.2%). The mean age of patients was 45.62 ± 17.32 yrs (range 16-75 yrs). Patients’ complaints are summarized in Table 1. No significant statistical difference was detected between males and females in terms of their chief complaints (P = 0.4). The most common late complications in patients were constipation in 9 cases (34.6%), pain in 9 cases (34.6%) and recurrence or existence of a mild (partial) prolapse in 7 cases (26.9%). None of the patients mentioned any sexual problem. 

**Table 1.** Frequency Distribution of Patients’ Chief Complaints

Chief complaint	Frequency	Percentage
**Constipation**	9	34.6
**Diarrhea**	1	3.8
**Bleeding**	3	11.5
**Fecal incontinence+ bleeding**	1	3.8
**Constipation+ bleeding**	6	23.1
**Pain**	1	3.8
**Constipation + fecal incontinence + bleeding**	2	7.7
**Constipation + fecal incontinence**	1	3.8
**Total**	24	92.3

The rate of constipation is higher than the rate reported by Corman it might be due to some cultural issues (i.e. not giving enough time for defecation, delay in visiting a physician and religious issues) or inappropriate nutrition. Incidence of bleeding in our study was also higher than expected. In our study, 4 patients (17%) had incontinence. Early complications such as infection, bleeding and anastomotic leak were not observed in any of our patients. The most common late complications reported were constipation (34.6%), pain (34.6%) and mild or partial prolapse (26.9%). Altemeier in his study on 106 cases reported 3 cases of recurrence (3). In Kimmins study, complications occurred in 10% of patients. But no morbidity or mortality was reported (4). In a study by Azimuddin, 16% of the patients relapsed during a follow up period of 50 months (6). In another study conducted in Minnesota, 10% recurrence was reported among 114 patients treated for rectal prolapse with Altemeier procedure (3).

In Brown study relapse was reported in 7 (5%) cases (7).The lowest rate of recurrence (1 case) was reported by Gopal. in their study on 18 middle aged patients (8). Our study results showed a higher rate of recurrence when compared to the above mentioned studies. However, the severity of prolapse recurrence was mild and the length of protruded segment was significantly smaller than the length before the operation and the majority of patients were mostly satisfied with the result. Their quality of life had improved and patients had better defecation. Although there is more recurrence rate than abdominal operation but Altemeier procedure can be safely performed for young males without risk of sexual dysfunction.
